# Novel measures of cardiovascular health and its association with prevalence and progression of age-related macular degeneration: the CHARM study

**DOI:** 10.1186/1471-2415-8-25

**Published:** 2008-12-22

**Authors:** Catherine A McCarty, Adam Dowrick, James Cameron, Barry McGrath, Luba D Robman, Peter Dimitrov, Gabriella Tikellis, Caroline Nicolas, John McNeil, Robyn Guymer

**Affiliations:** 1 Center for Human Genetics, Marshfield Clinic Research Foundation, Marshfield, MA, USA; 2 Centre for Eye Research Australia, University of Melbourne, East Melbourne, VIC, Australia; 3 Department of Epidemiology and Preventive Medicine, Monash University, Clayton, South Australia, Australia; 4 Department of Vascular Sciences, Monash University, Clayton, South Australia, Australia

## Abstract

**Background:**

To determine if novel measures of cardiovascular health are associated with prevalence or progression of age-related macular degeneration (AMD).

**Methods:**

Measures of the cardiovascular system: included intima media thickness (IMT), pulse wave velocity (PWV), systemic arterial compliance (SAC), carotid augmentation index (AI). For the prevalence study, hospital-based AMD cases and population-based age- and gender-matched controls with no signs of AMD in either eye were enrolled. For the progression component, participants with early AMD were recruited from two previous studies; cases were defined as progression in one or both eyes and controls were defined as no progression in either eye.

**Results:**

160 cases and 160 controls were included in the prevalence component. The upper two quartiles of SAC, implying good cardiovascular health, were significantly associated with increased risk of AMD (OR = 2.54, 95% CL = 1.29, 4.99). High PWV was associated with increased prevalent AMD. Progression was observed in 82 (32.3%) of the 254 subjects recruited for the progression component. Higher AI (worse cardiovascular function) was protective for AMD progression (OR = 0.30, 95%CL = 0.13, 0.69). Higher aortic PWV was associated with increased risk of AMD progression; the highest risk was seen with the second lowest velocity (OR = 6.22, 95% CL = 2.35, 16.46).

**Conclusion:**

The results were unexpected in that better cardiovascular health was associated with increased risk of prevalent AMD and progression. Inconsistent findings between the prevalence and progression components could be due to truly different disease etiologies or to spurious findings, as can occur with inherent biases in case control studies of prevalence. Further investigation of these non-invasive methods of characterizing the cardiovascular system should be undertaken as they may help to further elucidate the role of the cardiovascular system in the etiology of prevalent AMD and progression.

## Background

Age-related macular degeneration (AMD) is the leading cause of blindness in elderly Caucasians in the US [1], Australia [2] and other industrialized nations. The prevalence of AMD rises dramatically with age; 27% of people aged 90 and older have the more severe form of AMD [3]. With the anticipated increases in life expectancy, the incidence of AMD will necessarily increase. It is therefore important to identify risk factors for the disease with the hope of identifying strategies for primary prevention.

Other than age, the most consistent risk factor that has been identified for AMD is cigarette smoking [4]. It has been estimated that 20% of blindness in adults may be attributed to cigarette smoking [5]. Age and smoking are also known risk factors for the development of cardiovascular disease whilst other shared risk factors such as hypercholesterolemia, high fat intake, and high body mass index have been shown in some, but not all, studies to be associated with AMD [6].

There have been a number of reports from case-control and population-based cross-sectional studies on the association of cardiovascular disease parameters and AMD, with inconclusive results [7-12]. In the prospective Beaver Dam Eye Study, pulse pressure was modestly, but significantly, related to the five-year incidence of retinal pigment epithelial depigmentation and exudative macular degeneration [13] and higher pulse pressure and systolic blood pressure were also related to the 10-year incidence of exudative AMD [14]. Similar modest associations with pulse pressure, systolic blood pressure and the five-year incidence of AMD were observed in the Rottterdam Study [15].

A vascular model for AMD has been proposed in which lipid deposition in Bruch's membrane leads to increasing stiffness, increasing post-capillary resistance of the choroidal vasculature and elevated hydrostatic pressure of the chorio-capillaris [16]. It was suggested that this combination of high choroidal capillary pressure, breaks in calcified Bruch's membrane and vascular endothelial growth factor leads to choroidal neovascularization.

To further investigate the possible association of AMD with cardiovascular disease we took advantage of recent work in the field of cardiovascular disease where alternative, novel, non-invasive techniques are being proposed as robust measures of cardiovascular health. Whilst traditionally, brachial blood pressure (BP) has been used routinely as a screening test to assess central hypertension and risk of cardiovascular disease, it may not be the best prognostic parameter. Due to wave propagation phenomena and changing arterial wall composition, brachial BP is not identical to central aortic BP and it has been proposed that central BP may be more causal in terms of cardiovascular disease than brachial BP. Currently there is no overall method of assessment of global arterial function, but interest in this has been advanced by the availability of non-invasive measures of hemodynamic variables such as the arterial pressure waveform and volume flow. We wished to take advantage of these novel techniques to assess cardiovascular health in relation to prevalent AMD and also the likelihood of progression of early AMD to advanced disease.

Conduit artery (in particular aortic) mechanical properties are considered markers of vascular health, with increased stiffness considered an indicator of a more deleterious state. Indeed these changes in the arterial mechanical properties have been shown to bear a closer relationship to chronological age than many other parameters like graying of hair and loss of skin elasticity [17]. Thus it has been proposed that the assessment of the biological as opposed to the chronological age of an individual's arteries may be of use in the management and prevention of arterial disease. Large artery stiffness is known to increase with age even in the absence of overt cardiovascular disease. Aortic stiffness has a significant impact on central systolic BP and can be indirectly measured by several non-invasive methods that assess different aspects of large artery stiffness, such as pulse wave velocity (PWV), systemic arterial compliance (SAC) and carotid augmentation index (AI) [18-20].

With increasing intrinsic stiffening of elastic arteries there is a decrease in their buffering ability and therefore a resulting increase in pulse wave velocity (PWV). As central BP measurements are not routinely available, PWV can be considered an indirect marker. Specific studies have related PWV to cardiovascular and all-cause morbidity and mortality [20-25]. In the Cardiovascular Health and Age-Related Maculopathy (CHARM) study population we noted that brachial BP did not always predict PWV. Three percent of subjects assigned as hypertensive using brachial BP measurements had normal PWV whilst 18% of those with abnormally high PWV, considered to have true central hypertension, did not have abnormal brachial BP.

SAC reflects the function of an artery as a hollow receptacle in its ability to expand to accommodate the pulsatile blood flow [19] and associations have been found between arterial compliance and aortic stiffness with coronary artery disease [20]. SAC decreases as the arteries stiffen and has been shown to be inversely related to serum lipid levels [26].

Augmentation index (AI) is related to the timing of pressure wave reflection with regard to generated left ventricular systolic pressure. AI is positively correlated with PWV and BP, and inversely correlated with heart rate and height [24]. AI increases with age and sedentary lifestyle and is used as a surrogate measure of arterial stiffness.

Intima media thickness (IMT) quantification with B-mode ultrasonography is a non-invasive technique to identify and monitor preclinical atherosclerosis in large artery walls, especially the carotid [27-29]. Increased IMT is associated with increased risk of myocardial infarction and stoke.

To our knowledge, there have been no studies of the potential association of these novel cardiovascular health measurements in determining the risk of prevalent AMD or progression. Given that previous associations between AMD and CVD have been inconsistent we wondered if these newer measures of cardiovascular health would better define the association between these two diseases. Identification of modifiable risk factors for AMD would have important implications for the management of the disease prior to severe vision loss. The purpose of this study was to determine if cardiovascular health, as determined by these novel non-invasive techniques, was associated with prevalent AMD or AMD progression.

## Methods

A detailed description of the methodology for the CHARM (Cardiovascular Health &Age-Related Maculopathy) Study has been published previously [30]. Study procedures were approved by the Human Research and Ethics Committee at the Royal Victorian Eye and Ear Hospital (project number 99/372H) and all participants gave written informed consent prior to undergoing the examination. The research adhered to the tenets of the Declaration of Helsinki.

### Prevalent AMD Study Group

All participants with soft drusen or late stage AMD in the AMD progression study (see next section for details) were classified as cases for the study of risk factors for prevalent AMD. Fifty-one additional cases of late stage AMD were recruited from the Royal Victorian Eye and Ear Hospital and a private retinal clinic in Melbourne. Age- and gender-matched controls were recruited from the population-based Melbourne Visual Impairment Project (VIP) [2,3,31]. They had to be classified as controls at both the baseline and five-year follow-up examinations for the VIP to be classified as controls for the CHARM study. Subjects with only pigmentary changes or intermediate drusen were not included as either cases or controls. Subjects with only hard drusen were classified as controls.

### AMD Progression Study Group

Participants with documented AMD were recruited from two previous studies after their completion: the Vitamin E and Cataract and AMD (VECAT) Study [32-34] and the MelbourneVIP[2,3,31]. In both studies AMD was classified from dilated fundus photographs using the International Classification and Grading System [35]. Intermediate drusen were included in the definition of AMD, but small hard drusen in the absence of any other AMD features were not classified as AMD.

The VECAT study was a randomized clinical trial, conducted from 1994–1999, to assess the efficacy of 500 IU of vitamin E versus placebo in preventing the incidence and/or progression of cataract and AMD [31-33]. Community volunteers aged 55 to 80 years were recruited to participate in the five-year VECAT study. At baseline, soft drusen larger than 125 μm were found in 11.4% of the study cohort, retinal pigment epithelium changes in 9.5% and late stage AMD in 0.5% of participants [31]. The final results showed no effect of vitamin E supplementation on the incidence or progression of AMD [36], therefore there should be no bias in the assessment of AMD progression in this study cohort. Thus all of the participants with any signs of AMD, excluding bilateral end stage disease, were eligible for the CHARM Study.

The Melbourne Visual Impairment Project (VIP) was a population-based study of the distribution and determinants of eye disease in a random sample of permanent Melbourne residents aged 40 years and older [2,3,34]. Eligible residents were identified and recruited by household census from nine randomly selected pairs of census districts to participate in baseline assessments, conducted between 1992–1994. The prevalence of early age-related macular degeneration in the VIP was 14.9% in males and 15.2% in females, and the prevalence of late stage AMD was 0.58% in males and 0.76% in females [3]. All available VIP participants with AMD at baseline except those with bilateral endstage disease were eligible to participate in the CHARM study.

Recruitment for the CHARM Study commenced in 2000 and continued through 2002. Given the enrollment dates for the VECAT and VIP studies, the follow-up time interval for assessing AMD progression varied from six to ten years.

### Study Measures

The interviewer-administered questionnaire, based on the VIP questionnaire, contained questions about personal health history (including medication use), alcohol consumption, smoking history, ocular sunlight exposure, family history of AMD and blindness, quality of life, use of exogenous hormones, cognitive function, and visual function [34]. Dietary intake was assessed with a 4-page self-administered, semi-quantitative food frequency questionnaire [37]. Uniocular distance visual acuity was tested on a 4-meter retro-illuminated LogMAR chart and followed by objective refraction using Humphrey^® ^Autorefractor (Humphrey^® ^Instrument Inc, San Leandro, California) and subjective refinement to determine the best corrected visual acuity.

Height and weight were measured, as well as supine brachial blood pressure, measured with a Dinamap device (Critikon Vital Signs Monitor 1846 SX). Pulse pressure (the difference between the measured systolic and diastolic blood pressures) and heart rate were also measured. Carotid scanning for the assessment of intima media thickness (IMT) was assessed in supine subjects with a Toshiba SSH-140A imaging unit (Toshiba Corporation, Japan). Three longitudinal arterial images were taken at the right and left sides of the common carotid, bulb and internal carotid. The average IMT of the six far wall measurements, all 12 walls, and the mean maximum of all sites were calculated.

Details of the methods used to measure arterial structure and function are described below. The methods and the repeatability achieved by this group have been published previously [38].

Systemic arterial compliance (SAC) was estimated using the "area method" by obtaining a surrogate pressure waveform representing the aortic root driving pressure via applanation tonometry with a Millar Mikro-Tip pencil type transducer (Millar Instruments Inc, Houston, USA). Continuous wave Doppler velocimetry with a handheld instrument (Multi Dopplex II, Huntleigh Healthcare) was employed to assess flow in the ascending aorta. The mean of 10 waves was used to determine representative SAC.

Carotid pressure waveforms obtained by applanation tonometry were used to calculate the augmentation index (AI), defined as the difference between the first and second systolic peaks of the central arterial waveform, expressed as a percentage of the pulse pressure. The mean value from 10 pressure waves was taken as mean AI.

Pulse wave velocity (PWV) was measured by simultaneous recordings of arterial pressure waves at the right common carotid artery and the right femoral artery. Aortic PWV in meters per second was calculated as transit distance divided by transit time.

The dilated ophthalmic examination included a slit lamp examination and ophthalmoscopy with a 78 diopter lens. Signs of AMD were graded. Stereo photos of the macula were taken with a Zeiss FF4 camera. All the fundus photos from the CHARM examination as well as the baseline photos from the VIP and VECAT studies were graded by a single trained, experienced photo grader. Ten percent of the cohort was graded by another senior grader to ensure consistency in grading. Any difficult photos were assessed by the graders as well as a retinal specialist (RG).

The presence of AMD features was graded according to the International Classification System [35,40]. Six levels of AMD were scored: 1) no drusen or hard drusen only (none in our study cohort), 2) intermediate drusen AND/OR hyperpigmentation without hypopigmentation, 3) distinct or indistinct soft drusen OR hypopigmentation with or without hyperpigmentation, 4) distinct or indistinct soft drusen AND hyper- or hypopigmentation, 5) geographic atrophy, 6) neovascular AMD. The presence of small hard drusen with no other features present was not included in the definition of AMD. An increase in AMD severity from level 2, 3 or 4 at baseline to one or more levels in either eye, and also an increase of two or more steps in the specific grades (as defined by the International Classification and Grading System) [35,40] used to assess size, total number, area occupied by a lesion and spread to a more central location within a level, were defined as progression. A comparison of the results of grading from the three different cameras used in the VIP, VECAT and CHARM studies revealed good agreement, with kappa values ranging from 0.69 to 0.90 for the various AMD features [39,40].

Fasting blood samples were analyzed for blood glucose, total blood cholesterol, high density lipoprotein (HDL) cholesterol, low density lipoprotein (LDL) cholesterol, fibrinogen, IgA, IgM, total white cells, lymphocytes, and apolipoprotein E. Genomic DNA was isolated from venous blood leukocytes using a standard phenol/chloroform extraction procedure [41]. The molecular techniques used to genotype and score the ε2, ε3 and ε4 alleles of the APOE gene have been described elsewhere [42]. Presence of the ε4 allele was included as a covariate in the multivariate models because of its proven association with cardiovascular disease and its protective effect on AMD which has been shown in a number of studies [22,23]. We have reported on the APOE gene effect on the CHARM cohort where we found the ε4 allele to be protective against progression of AMD [42].

Interview data were entered directly into a Microsoft Access database that contained internal consistency checks as well as confirmation of responses given during previous interviews. All other data were entered twice and verified. Data were analyzed with SPSS^®^, version 15.0. Univariate analyses consisted of chi-squared and t-tests. Because frequency matching was used for controls rather than one-to-one matching, simple logistic regression was employed rather than conditional logistic regression. Multivariable adjusted logistic regression models and analysis of co-variance models were also conducted. A p-value < 0.05 was considered statistically significant. Multivariable adjusted logistic regression models were employed to evaluate the relationship of augmentation index (AI), intima media thickness (IMT) and systemic arterial compliance (SAC) to prevalent AMD. All models included 'ever smoked' because of its known association with AMD prevalence and cardiovascular risk factors and outcomes, and mean arterial pressure because it was correlated with all of the independent variables (correlation coefficients ranged from 0.10 to 0.44, p-values ranged from 0.10 to < 0.001). The presence of an Apoε4 allele was included because it has been shown to increase risk of CHD and has been shown to be associated with AMD in numerous studies. Other covariates were included in sensitivity analyses because they were found to be significantly correlated with the independent variables of interest. Multivariable adjusted logistic regression analyses were employed to assess the independent relationship between four significant/borderline significant factors and AMD progression. SAC was also included in a sensitivity analysis because of the significant result observed with prevalent AMD. Age, 'ever smoked' and Apoε4 genotype were included in all multivariate models. Other co-variates that were included in the models for sensitivity were selected because they were significantly correlated with the independent variable of interest.

## Results

### Cardiovascular Disease and Prevalent AMD

There were 320 subjects available for the analysis of risk factors for prevalent AMD (160 cases and 160 age- and gender-matched controls). The frequency matching produced equivalent groups for analysis, as evidenced by non-significant differences in demographic factors between the two groups. The mean age of the study subjects was 73.9 years (range 52–93 years) and 40.9% were male. The 160 population-based controls included subjects with no fundus abnormalities (n = 16) and many subjects with hard drusen only (n = 136). Of the 160 AMD cases, 26% had late stage AMD (geographic atrophy or neovascular AMD) in one eye, whilst the remaining (74.0%) had soft drusen in at least one eye.

Nearly 12% (n = 18) of the AMD cases reported a history of AMD in their parents or siblings, compared with 2% (n = 3) of controls (chi-squared = 12.04, p = 0.001). AMD cases not more likely then controls to have ever smoked (49.0% versus 44.6%, chi-squared = 0.63, p = 0.43). Descriptive characteristics of the continuous cardiovascular measures and risk factors in cases and controls are summarized in Table 1. In the univariate analyses of variables that are risk factors for CVD or were measures of cardiovascular health, a lower AI, indicating healthier cardiovascular function was found to be significantly associated with AMD. (t = -1.93, p = 0.05).

**Table 1:** Univariate comparison of cardiovascular measures and risk factors in prevalent AMD cases and controls

**Variable**	**AMD cases Mean (SD)**	**Controls Mean (SD)**	**t-test, p-value**
Systemic arterial compliance (log)	-0.58 (0.29)	-0.63 (0.29)	1.605, 0.11
Augmentation index	15.43 (9.22)	17.60 (10.23)	-1.93, 0.05
Pulse wave velocity, aortic	12.28 (4.15)	11.80 (4.16)	1.00, 0.32
Average IMT of 12 walls (log)	-1.09 (0.066)	-1.08 (0.060)	-0.77, 0.44
Mean maximum IMT (log)	-0.95 (0.081)	-0.95 (0.072)	0.24, 0.81
Mean arterial blood pressure	100.4 (13.0)	101.0 (15.3)	-0.39, 0.70
Total cholesterol	5.57 (1.01)	5.62 (1.03)	-0.38, 0.70
HDL cholesterol	1.62 (0.48)	1.59 (0.50)	0.505, 0.61
LDL cholesterol	3.32 (0.87)	3.35 (0.92)	-0.33, 0.74
Triglycerides (log)	0.22 (0.46)	0.25 (0.48)	-0.60, 0.55
Heart rate	61.6 (10.1)	63.4 (11.0)	-1.49, 0.14
Glucose (log)	1.66 (0.18)	1.66 (0.23)	0.06, 0.95
Fibrinogen	4.35 (0.88)	4.22 (0.94)	1.23, 0.22

In multivariate models adjusted for APOE4 allele and having ever smoked (Table 2), the upper two quartiles of SAC, implying good cardiovascular function, were significantly associated with an increase in prevalent AMD (OR = 2.04, 95%CL = 1.05, 3.95 for quartile 3 and OR = 2.54, 95% CL = 1.29, 4.99 for quartile 4) and the test for linear trend between SAC and prevalent AMD was statistically significant (Mantel Haenszel chi-squared = 4.73, p = 0.03). Although not statistically significant, mean maximum IMT, also showed a trend indicating that thicker, more diseased vessels might protect from AMD. This protection from AMD was also seen as a trend when the worst AI quartile was compared to the best, but was not consistent across all quartiles. Whilst a greater aortic PWV, indicative of worsening risk of cardiovascular disease, was correlated with increased risk of AMD, the greatest risk was in the second lowest quartile, with risk then becoming relatively less for the upper (worst) two quartiles of PWV. Sensitivity analyses that incorporated other vascular factors associated with the independent variables of interest (glucose, cholesterol, heart rate, mean arterial pressure) did not substantially alter these associations (data not shown). The same trends reported in Table 2 were observed when restricting the analysis to only the late stage AMD cases, although these findings were not statistically significant due to the smaller number of cases. As an example, for SAC, the multivariate odds ratios were 1.23 (95% CL = 0.44, 3.44), 1.17 (95%CL = 0.41, 3.28) and 1.30 (95% CL+0.43, 3.96) respectively for the three upper quartiles in comparison with the lowest quartile.

**Table 2:** Multiple logistic regression analyses of cardiovascular risk factors and risk of AMD prevalence adjusted for ever having smoked and APOE4 allele status.

**Cardiovascular risk factor**	**Number (%) of cases**	**Odds ratio**	**95% CL**
Systemic arterial compliance*			
Quartile 1	30 (19.4%)	1.0	
Quartile 2	39 (25.2%)	1.92	0.09, 3.76
**Quartile 3**	42 (27.1%)	**2.04**	**1.05, 3.95**
**Quartile 4**	44 (28.4%)	**2.54**	**1.29, 4.99**
Augmentation index			
Quartile 1	36 (23.4%)	1.0	
Quartile 2	36 (23.4%)	0.71	0.37, 1.35
Quartile 3	41 (26.6%)	1.03	0.55, 1.90
Quartile 4	41 (26.6%)	0.60	0.33, 1.09
Pulse wave velocity (aortic)			
Quartile 1	32 (21.3%)	1.0	
Quartile 2	37 (24.7%)	1.60	0.81, 3.15
**Quartile 3**	41 (27.3%)	**2.03**	**1.01, 4.09**
Quartile 4	40 (26.7%)	1.84	0.88, 3.86
Mean maximum IMT			
Quartile 1	40 (25.3%)	1.0	
Quartile 2	37 (23.4%)	0.79	0.42, 1.50
Quartile 3	40 (25.3%)	0.94	0.49, 1.78
Quartile 4	41 (25.9%)	0.91	0.48, 1.74

*p-value for linear trend = 0.03

(significant factors in bold)

### Cardiovascular Disease and AMD Progression

In the cohort of 254 subjects recruited for the progression component of the study, AMD progression was observed in 82 (32.3%) individuals. The mean age of the subjects whose AMD progressed was 76.0 years, compared with 73.1 for those subjects whose AMD did not progress (t-test = -2.96, p = 0.004). The gender distribution was not significantly different between progressed (47.1% male) cases and non-progressed (46.3% male) controls (chi-squared = 0.13, p = 0.911). Subjects whose AMD had progressed were significantly more likely to have ever been smokers (59.8% versus 40.7%, chi-squared = 8.1, p = 0.004) and were significantly more likely to have a first degree relative with AMD (9.9% versus 2.4%, Fisher's exact p-value = 0.022).

Unadjusted comparisons of cardiovascular risk factors revealed frankly significant or borderline significant differences in AI (t = 2.576, p = 0.011), PWV (t = -1.53, p = 0.128), and mean maximum IMT (t = -1.40, p = 0.165) between AMD progressors and non-progressors (Table 3).

**Table 3:** Univariate comparison of cardiovascular measures and risk factors in AMD progressors and non-progressors

**Variable**	**AMD progression Mean (SD)**	**No AMD progression Mean (SD)**	**t-test, p-value**
Systemic arterial compliance (log)	0.30 (0.49)	0.29 (0.51)	-0.583, 0.56
Augmentation index	13.57 (8.44)	16.71 (9.77)	2.576, 0.01
Pulse wave velocity, aortic	12.51 (4.24)	11.64 (3.95)	-1.53, 0.13
Average IMT of 12 walls (log)	0.08 (1.16)	0.08 (1.17)	-0.491, 0.62
Mean maximum IMT (log)	0.12 (1.20)	0.11 (1.21)	-1.396, 0.17
Mean arterial blood pressure	99.83 (12.78)	99.93 (12.04)	0.06, 0.95
Total cholesterol	5.55 (0.91)	5.50 (1.00)	-0.319, 0.75
HDL cholesterol	1.57 (0.44)	1.54 (0.45)	-0.607, 0.55
LDL cholesterol	3.31 (0.80)	3.34 (0.83)	0.285, 0.78
Triglycerides (log)	1.33 (1.61)	1.29 (1.62)	-0.426, 0.67
Heart rate	64.1 (9.01)	62.3 (10.74)	-1.39, 0.17
Glucose (log)	5.31 (1.22)	5.16 (1.19)	-1.127, 0.26
Fibrinogen	4.20 (0.86)	4.12 (0.76)	-0.715, 0.48

After adjusting for age, ever having smoked and APOE4 allele status, a higher AI, or worsening cardiovascular function was associated with reduced risk of AMD progression (OR = 0.54, 0.31 and 0.30 for the upper three quartiles respectively in comparison with the lowest quartile, Table 4). The aortic PWV results were similar to the prevalence data. Risk of progression was associated with the second lowest (or second healthiest) quartile of PWV, becoming less as the PVW got progressively worse. (OR 6.22 in second quartile, compared to an OR of 3.36 in the highest quartile). There were no significant associations found between mean maximum IMT, SAC and risk of AMD progression. Sensitivity analyses that included cardiovascular factors such as cholesterol levels, glucose, heart rate and mean arterial pressure, known to be associated with the independent variables of interest did not markedly change the results (data not shown).

**Table 4:** Multiple logistic regression analyses of cardiovascular risk factors and risk of AMD progression adjusted for age, APOE4 status and having ever smoked.

**Cardiovascular risk factor**	**Number (%) of cases**	**Odds ratio**	**95% CL**
Systemic arterial compliance			
Quartile 1	18 (21.7%)	1.0	
Quartile 2	20 (25.2%)	1.18	0.53, 2.61
Quartile 3	18 (21.7%)	1.13	0.50, 2.55
Quartile 4	23 (27.7%)	1.80	0.80, 4.04
Augmentation index			
Quartile 1	30 (38.4%)	1.0	
Quartile 2	21 (26.9%)	0.54	0.26, 1.16
**Quartile 3**	14 (17.9%)	**0.31**	**0.14, 0.70**
**Quartile 4**	13 (16.7%)	**0.30**	**0.13, 0.69**
Pulse wave velocity (aortic)*			
Quartile 1	7 (9.1%)	1.0	
**Quartile 2**	26 (33.8%)	**6.22**	**2.35, 16.46**
**Quartile 3**	21 (27.3%)	**3.69**	**1.38, 9.89**
**Quartile 4**	23 (29.9%)	**3.36**	**1.24, 9.06**
Mean maximum IMT			
Quartile 1	14 (17.1%)	1.0	
Quartile 2	19 (23.2%)	1.26	0.54, 2.92
Quartile 3	25 (30.5%)	1.77	0.78, 4.01
Quartile 4	24 (29.3%)	1.34	0.57, 3.17

*p for linear trend = 0.008, **p for linear trend = 0.04

(significant factors in bold)

## Discussion

A number of studies have assessed the potential relationship between traditional cardiovascular risk factors and AMD prevalence, with primarily weak and inconsistent findings [6]. We were unable to find an association between traditional brachial blood pressure measurements and either the prevalent or progressive AMD in the present study. In this study we also sought to determine whether novel, potentially more robust, methods to assess the cardiovascular system might give more insight into the relationship between cardiovascular health and AMD. AI is determined by the magnitude and relative arrival time of the reflected pressure wave relative to cardiac ejection. As such it is determined by distance to the distal reflecting site, itself affected by height and state of the peripheral vasodilation as well as heart rate and average PWV in the aorta. SAC provides the best estimate of cardiac afterload and (probably) of coronary perfusion efficiency. PWV has been shown to be the most robust indicator of overall atherosclerotic burden and cardiovascular risk. We know of no other study that has quantified arterial structure and function using these novel measures of cardiovascular health in relation to both prevalent and progressive AMD. Contrary to expectations, this study has produced some significant findings that would suggest that individuals with healthier cardiovascular function are more at risk of not only developing AMD, but also progressing to more severe stages.

Individuals with greater systemic arterial compliance (SAC), one indicator of better cardiovascular health, had a higher rate of prevalent AMD. People with higher AI (implying stiffer arteries), were less likely to show progression of their AMD. The results of the PVW in both the prevalence and progression cohort showed similar interesting results. Whilst the lowest PWV (indicative of less cardiovascular disease) did appear to be associated with a higher rate of prevalent AMD and a greater likelihood to progress, as PWV got progressively greater, the increased risk seen in the second best quartile of PWV lessened. Considering that these cohorts, particularly the progression cohort were healthy volunteers, we may have seen this trend continue if people with worse CVD were included.

This same CHARM cohort has been assessed to find risks for progression that are consistent with the literature in terms of age, smoking, and the APOE gene risk [41]. We are therefore confident that our cohort and our analysis of AMD and its progression are robust. These surprising findings may go some way towards explaining the inconsistencies found in the numerous studies reporting on cardiovascular disease and AMD. Of course, we cannot rule out the possibility that the findings are spurious. Although this was a study of progression and prevalent AMD, comparison of results from studies of incidence and prevalence often result in discrepant findings, potentially because of the many potential biases (such as recall bias) inherent in case/control studies of prevalent disease. Also, prevalent cases likely have better cardiovascular health than the general population because they were still alive to participate in the study. Although the participation rates of the two source studies, the VECAT and the VIP, were quite high, it is possible that the eye and/or cardiovascular health of the participants were significantly different to the non-participants. We have no data about the non-participants for comparison to assess this potential bias. Another potential bias and limitation in our study design is the fact that the cardiovascular measures were done at follow-up for the progression component of the study. Subjects with poorer cardiovascular health could have died between the baseline assessment of AMD status and the follow-up visit to categorize AMD progression and cardiovascular health. These potential biases and limitations may all have affected the results to some degree that is not possible to quantify.

Based on our results, we hypothesize that a reasonably healthy cardiovascular system is necessary to drive the processes that lead to the development and progression of AMD. This would be consistent with the general clinical observation that most people with AMD do not appear to be vasculopaths. This concept is not new in retinal diseases, for in diabetic retinopathy an eye is protected by carotid disease, and is put at increased risk when the carotid disease is repaired at surgery [47,48]. The counterintuitive finding of a protective effect of poorer cardiovascular health on the prevalent AMD and progression of AMD has some parallels in the genetics of AMD where the Apo e4 allele has been shown in numerous studies to be protective for AMD, yet is a risk factor for CVD and CVD mortality.

Our non-significant findings for a potential relationship between IMT and AMD are similar to those observed in the Cardiovascular Health Study and differ from the 2.5-fold increased risk of prevalent AMD that was found with common carotid plaques in the Rotterdam Study. In this latter study, carotid wall thickness was also found to be significantly associated with incident AMD, although the association was much weaker (OR = 1.15) and therefore of questionable clinical significance. Those with plaques (advanced disease) had increased rate of prevalent AMD, whilst those with intermediate stages of CV disease (increased IMT without plaque) had increased incidence. This is compatible with our findings for incidence (SAC, PWV) and prevalence (PWV, AI).

Our results, whilst initially surprising, might be explained as follows if they are true. An individual requires reasonable cardiovascular health (higher SAC, lower AI, lower PWV) to be susceptible to AMD development. Development and progression may commence alongside worsening arterial function (i.e. increasing AI, PWV and decreasing SAC). It is well known that in survivors, arterial stiffness plateaus with age, consistent with irreversible structural changes. Our results suggest that those who have reached this stage of maximal stiffening are protected from both incident cases in those that do not already have AMD, or further progression if they already have the disease.

The elderly with high SAC were more likely to have AMD. The association of a requirement for good indices of cardiovascular function to get AMD may be a result of those with such indices surviving long enough, or may be indicative of a direct causal effect. The first quartile of the PWV group, those who are most "well" in a cardiovascular risk sense, do not exhibit significant systemic atherosclerosis. Moderate stiffening (second quartile group) are those with worsening arterial function but does not include those individuals who have already achieved their "worst state". Those in the fourth quartile have plateaued and can get no stiffer in terms of arterial structure-function.

## Conclusion

Further investigation of these novel, non invasive techniques of assessing the cardiovascular system seems warranted as they may not only uncover further modifiable risk factors, but may go some way to increase our understanding of the pathogenesis of AMD and its progression.

## Authors' contributions

CAM conceived the study, obtained grant funding, analyzed the data and drafted the manuscript. AD collected the carotid artery data, interpreted the carotid artery results, reviewed and approved the final manuscript. JM supervised the carotid artery measurements and interpretation, drafted sections of the manuscript, and reviewed and approved the final manuscript. BM supervised the carotid artery measurements and interpretation, drafted sections of the manuscript, and reviewed and approved the final manuscript. LDR assisted in study design, conducted the ophthalmic assessments, assisted in the statistical analysis, drafted sections of the manuscript and reviewed and approved the final manuscript. PD collected the orthoptic data, and reviewed and approved the final manuscript. GT grade and interpreted all of the fundus photos, assisted in the statistical analysis and reviewed and proved the final manuscript. CN recruited subjects, collected all of the demographic and risk factor data, and reviewed and approved the final manuscript. JM assisted in the overall study design, the acquisition of study funding and reviewed and approved the final manuscript. RG supervised study staff, acquired study funding, assisted in clinic subject recruitment, interpreted data, and assisted in drafting the manuscript.

## Pre-publication history

The pre-publication history for this paper can be accessed here:


